# Feelings of emptiness, pathological personality traits, and suicidal risk: an exploratory study using the modified subjective emptiness scale in an Italian general population sample

**DOI:** 10.3389/fpsyg.2026.1748134

**Published:** 2026-07-13

**Authors:** Marta Moselli, Arianna Quassoni, Alessandra D’Agostino

**Affiliations:** 1 Department of Clinical, Dynamic and Health Psychology; “la Sapienza” University of Rome, Rome, Italy; 2 Department of Humanities (DISTUM), University of Urbino Carlo Bo, Urbino, Italy

**Keywords:** AMPD, confirmatory factor analysis, C-SSRS, mediation model, personality functioning, PID-5, subjective emptiness, suicidality

## Abstract

**Introduction:**

Subjective emptiness is a painful affective experience increasingly recognized as transdiagnostic and multidimensional. Yet, empirical assessment of its multidimensional nature remains limited. Because emptiness stems from instability in self-organization and emotional integration, it is closely connected to personality functioning. Previous studies found that identity pathology and emptiness independently predicted suicidal ideation, with emptiness partially mediating the relationship between identity disturbance and suicidality. This study developed and validated the Modified Subjective Emptiness Scale (M-SES), a 25-item instrument designed to capture the phenomenological facets of emptiness and examined its associations with maladaptive personality traits and suicidal ideation in a community sample.

**Methods:**

Participants were 202 adults (71.3% female; *M* = 41.2, SD = 14.4) who completed the M-SES, PID-5-SF, BSI, and C-SSRS. Confirmatory factor analysis tested the hypothesized four-factor structure. Reliability, convergent validity, and mediation analyses were performed using standard indices.

**Results:**

Confirmatory factor analysis supported a four-factor structure—Physical/Bodily emptiness, Interpersonal/Social disconnection, Dissatisfaction/Lack of purpose, and Severity/Self-level impairment—showing good fit indices. A second-order factor model confirmed that the four dimensions load on a higher-order Subjective Emptiness factor. All M-SES dimensions correlated strongly with Negative Affectivity and Detachment, and moderately with Psychoticism, supporting convergent validity. Regression analyses showed that Interpersonal/Social disconnection uniquely predicted suicidal ideation severity. Mediation analyses revealed significant indirect effects of Detachment and Psychoticism on suicidal ideation via interpersonal emptiness, while Disinhibition exerted only a direct effect.

**Discussion:**

Findings support the M-SES as a psychometrically sound measure capturing both the structural and affective dimensions of subjective emptiness. Interpersonal emptiness emerged as a central experiential pathway linking maladaptive personality traits to suicidal ideation, emphasizing its clinical relevance as a target for assessment and intervention. Future studies should extend validation to clinical populations and longitudinal designs.

## 1 Introduction

Subjective emptiness is a complex and painful affective experience characterized by a pervasive sense of inner absence, disconnection in internal experience, loss of meaning, absence of vitality and continuity in self-perception, and detachment from the self and others (D’Agostino et al., 2020; Hein et al., 2025; Herron and Sani, 2022). In the literature, emptiness has been described as both a feeling state and a structural alteration in self-experience (Zandersen and Parnas, 2019). Phenomenological authors have interpreted emptiness as a disturbance in self-affection and continuity (Parnas et al., 2005; Stephensen et al., 2024), whereas psychodynamic models describe it as the emotional counterpart of identity diffusion and an impaired ability to maintain internal representations (Kernberg, 1975). Contemporary clinical and phenomenological literature point to the presence of three domains representing the feeling of emptiness: (a) Physical/bodily emptiness, reflecting sensations of an internal void or loss of vitality; (b) Interpersonal/social disconnection, reflecting feelings of isolation, alienation, and loss of relational meaning; (c) Dissatisfaction/lack of purpose, referring to an existential sense of futility and meaninglessness (D’Agostino et al., 2020)-

Historically, chronic feelings of emptiness have been considered a hallmark of borderline personality disorder (BPD) (Hein et al., 2025; Klonsky, 2008), and a sense of emptiness still is one of the diagnostic criteria for BPD (Pompili et al., 2023). However, emptiness is not limited to borderline pathology but appears across a range of psychiatric conditions, including depressive, narcissistic, dissociative, and psychotic disorders, and even in non-clinical populations (D’Agostino et al., 2021; Hopwood and Gjorgjieva, 2024). Emptiness thus represents a transdiagnostic phenomenon (Konjusha et al., 2021) that could be viewed as a general marker of personality and affective dysfunction. A longitudinal investigation further showed that the severity of emptiness predicts poor functional outcomes and persistent suicidal ideation, even when controlling for affective instability and depressive symptoms (Hein et al., 2025). The experience of emptiness as a negative condition may also be culturally determined. Indeed, there are cultural traditions in which emptiness can assume positive connotations and is even actively sought as a state of balance or spiritual clarity (Nakamura and Carta, 2021). However, these considerations lie beyond the scope of the present study.

Because emptiness stems from instability in self-organization and emotional integration, it is closely connected to personality functioning. This connection is particularly evident when emptiness is conceptualized within a dimensional framework, such as the Alternative Model for Personality Disorder (AMPD). The AMPD emphasizes impairments in self- and interpersonal functioning as the core of personality pathology (Sharp and Wall, 2021). In the DSM-5-TR AMPD (Hopwood et al., 2011; Nussbaum, 2022), the domains of negative affectivity, detachment, and psychoticism capture fundamental vulnerabilities that may underlie the experience of emptiness. Individuals high in negative affectivity may experience emptiness as pervasive despair; those high in detachment, as alienation and loss of connection; and those high in psychoticism, as fragmentation and unreality (Hopwood and Gjorgjieva, 2024). Similarly, narcissistic pathology has long been associated with emptiness as a defensive reaction to unmodulated shame and dependence on external validation (Ronningstam, 2010).

Suicide is one of the leading causes of preventable death worldwide. However, most predictive models based on psychiatric diagnoses or static risk factors demonstrate poor accuracy (Franklin et al., 2017). These approaches fail to capture the dynamic processes that transform psychological pain into suicidal intent. Consequently, current theories conceptualize suicidality as a multistage process rather than a discrete event or symptom (Barzilay and Apter, 2014). Painful affective states, such as feelings of emptiness, can precipitate a condition of unbearable psychological pain, starting with the experience of intense emotional distress (psychache; Shneidman, 1993). When this pain becomes intolerable and available regulatory strategies fail, individuals may attempt to restore coherence and control through suicidal ideation (Casini et al., 2024; Williams et al., 2021). The experience, meaning, and management of this suffering are profoundly shaped by personality functioning (Hatkevich et al., 2019; Williams et al., 2023). Meddaoui et al. (2025) found that identity pathology and emptiness independently predicted suicidal ideation, with emptiness partially mediating the relationship between identity disturbance and suicidality. Similarly, D’Agostino et al. (2021) reported that emptiness correlated with suicidal thoughts in community and clinical samples.

From this perspective, subjective emptiness is a critical node in the emotional landscape of the suicidal process. It serves as an expression of personality vulnerability and a potential mediator between pathological personality traits and suicidal ideation. Investigating this relationship in the general population could help elucidate the early mechanisms through which personality dysfunction translates into suicidal risk and identifies potential targets for prevention and intervention.

## 2 Aims

Despite the growing recognition of subjective emptiness as a clinically and theoretically relevant phenomenon, empirical research remains limited. Existing measures capture emptiness as a unidimensional construct, frequently conflating it with depressive or dissociative features. In fact, the only two English-language measures currently available for assessing subjective emptiness are the *Experienced Levels of Emptiness Scale* (Hazell, 1984), which was not developed for clinical purposes and focuses exclusively on existential dimensions, and the *Subjective Emptiness Scale* (SES; Price et al., 2022). This neglects both the phenomenological complexity of subjective emptiness and its relationship with personality functioning. As a result, the development of reliable instruments capable of investigating the role of emptiness in personality pathology and suicidal processes has been significantly hindered.

Building on this literature, the Italian SES-I represented an initial psychometrically sound operationalization of subjective emptiness in the Italian context. However, its unidimensional structure did not allow for differentiation among the bodily, interpersonal, existential, and self-structural manifestations of emptiness, while its strong correlations with depression and dysphoria raised concerns regarding the construct’s discriminant validity. The present study aimed to address this limitation by developing and validating the Modified Subjective Emptiness Scale (M-SES), a revised and expanded version of the Italian Subjective Emptiness Scale (SES-I; D’Agostino et al., 2021). The M-SES assesses the multidimensional structure of emptiness and encompasses four domains derived from contemporary clinical and phenomenological literature: (a) Physical/bodily emptiness; (b) Interpersonal/social disconnection; (c) Dissatisfaction/lack of purpose; (d) Severity/self-level impairment, indicating the degree of disturbance in self-coherence and identity organization

In line with the proposed theoretical framework, the study had three main objectives:

1. Create and examine the factorial structure and psychometric properties of the M-SES in a general population sample by testing its internal consistency, dimensionality, and construct validity.
2. Explore the associations between the different dimensions of emptiness and pathological personality traits, as defined by the DSM-5 Alternative Model for Personality Disorders (PID-5-SF domains: Negative Affectivity, Detachment, Antagonism, Disinhibition, and Psychoticism) and indicators of suicidality (as measured by the C-SSRS).
3. Test a mediation model in which specific dimensions of emptiness mediate the relationship between pathological personality traits and the severity of suicidal ideation.

## 3 Methods

### 3.1 Developing the M-SES

The Modified Subjective Emptiness Scale (M-SES) is a 27-item self-report instrument designed to explore the subjective experience of emptiness.

It originates from the Subjective Emptiness Scale-Italian version (SES-I; D’Agostino et al., 2021), a unidimensional tool that provided a first operationalization of the subjective experience of emptiness, capturing its global intensity as an affective state. The SES-I showed good internal consistency and convergent validity, but its single-factor structure did not allow for a nuanced representation of the different experiential configurations of emptiness, and showed strong correlations with other constructs, e.g., depression, dysphoria, raising questions on the validity of the construct. Building on the theoretical and clinical review by D’Agostino et al. (2020), emptiness was progressively reconceptualized as a multidimensional phenomenon involving partially distinct experiential domains rather than a single homogeneous state. More specifically, the review highlighted that phenomenological, psychodynamic, and clinical accounts of emptiness consistently converged around three recurrent experiential configurations.

The first concerned Physical/Bodily emptiness, characterized by experiences of inner void, loss of vitality, emotional numbness, and disconnection from bodily feeling states. The second involved Interpersonal/Social disconnection, referring to alienation, loneliness, loss of relational meaning, and the subjective impossibility of emotional connection with others. The third domain, Dissatisfaction/Lack of purpose, reflected existential feelings of incompleteness, purposelessness, futility, and lack of direction or fulfillment. These dimensions were subsequently operationalized into item content intended to capture their characteristic experiential and phenomenological qualities, translating recurrent clinical descriptions of emptiness into first-person self-report statements.

During the revision process, it also became evident that certain experiences of emptiness involved not only affective or interpersonal suffering, but more profound disturbances in the organization of self-experience itself. Accordingly, a fourth domain, Severity/Self-level impairment, was introduced to capture alterations in self-coherence, identity continuity, and the basic sense of subjectivity.

Items for this scale were derived from both phenomenological and psychodynamic sources—specifically, the Examination of Anomalous Self-Experience (EASE; Parnas et al., 2005) and the Structured Interview of Personality Organization (STIPO; Clarkin et al., 2004). From the EASE, three critical items (4, 10, and 26) were adapted to capture experiences characteristic of basic self-disorders, particularly the phenomenon of “Anderssein”—a disturbance in the first-personal givenness of experience, in which the individual perceives their own mental life as alien or not truly belonging to themselves. This dimension distinguishes basic self-disturbance (a structural alteration of subjectivity) from narrative self-disturbance, which emerges at a more social and linguistic level of identity. The final version of the M-SES comprises 27 items, rated on a 5-point Likert scale (0 = “not at all true for me” – 4 = “completely true for me”).

### 3.2 Other measures

Personality Inventory for DSM-5-Short Form (PID-5-SF; Somma et al., 2019): The PID-5-SF (Italian translation) is a 100-item measure assessing the five pathological personality domains proposed by the DSM-5 Alternative Model for Personality Disorders (AMPD): Negative Affectivity, Detachment, Antagonism, Disinhibition, and Psychoticism. Responses are rated on a 4-point Likert scale (0 = “very false or often false” to 3 = “very true or often true”).

Columbia–Suicide Severity Rating Scale (C-SSRS; Posner et al., 2011): The C-SSRS is a semi-structured interview that measures suicide ideation and behavior. The C-SSRS allows rates to obtain scores on four dimensions: (a) the suicidal Ideation scale, which is measured on a 5 points Likert scale (from 1 = desire to be dead to 5 = suicide intent with a plan) assessing the severity of the ideation; (b) the Intensity of the Ideation scale, which is comprised of five items (i.e., frequency, duration, controllability, deterrents, reasons for ideation), each rated on an ordinal scale (total scores ranging from 2 to 25); (c) the Suicide Behavior scale, which focused on four types of suicidal behaviors; (d) the Behavior Lethality subscale. The C-SSRS was translated in more than 140 country-specific languages, including Italian.^1^

Brief Symptom Inventory (BSI; De Leo et al., 1993): The BSI (Italian translation) is a 53-item self-report inventory derived from the SCL-90-R that assesses current psychological distress across nine symptom dimensions: Somatization, Obsession–Compulsion, Interpersonal Sensitivity, Depression, Anxiety, Hostility, Phobic Anxiety, Paranoid Ideation, and Psychoticism, as well as the Global Severity Index (GSI) as a measure of overall distress. Each item is rated from 0 (“not at all”) to 4 (“extremely”).

Demographic and clinical variables: Age, assigned sex at birth, family history of psychiatric disorder, and prior psychological or psychiatric treatment were collected for descriptive and control purposes.

### 3.3 Participant

The sample consisted of 207 participants (71.3% female, 28.7% male), aged between 20 and 78 years (*M* = 41.2, SD = 14.4). Participants were recruited through online advertisements and university mailing lists. Average completion time was approximately 25 min. Missing data were minimal (<3%) and handled through listwise deletion for analyses requiring complete cases. Inclusion criteria were age ≥ 18 and proficiency in Italian. Exclusion criteria included: (a) the presence of medical and/or neurological conditions (e.g., disabling degenerative diseases); (b) acute psychotic symptoms; (c) intellectual disability (IQ < 80); (d) poor knowledge of the Italian language; and (e) any other condition that could impair comprehension or the ability to complete the interview and questionnaires. Participation was voluntary and anonymous, and all participants provided informed consent in accordance with the Declaration of Helsinki. The study protocol was approved by the institutional ethics committee (Comitato Etico per la Sperimentazione Umana (CESU) Estratto del Verbale seduta n. 93 del 24 aprile 2025).

### 3.4 Data analysis

All analyses were conducted using Jamovi (version 2.4.11) (Gallucci, 2020).

Reliability was evaluated using Cronbach’s α and McDonald’s ω coefficients.

Confirmatory Factor Analysis (CFA) tested the four-factor model identified through PCA. Model fit was assessed via χ^2^, RMSEA, CFI, TLI, and SRMR indices.

Convergent and discriminant validity were examined through Pearson correlations between M-SES subscales, PID-5-SF domains, BSI domains, and C-SSRS ideation severity. Participants were also assessed for previous suicide attempts. However, because the study was conducted in a non-clinical population, the number of participants reporting previous suicide attempts was too small (5.3%) to allow for adequate statistical analysis. Therefore, the main outcome measure of suicidality considered in the present study was the severity of suicidal ideation, as assessed by the C-SSRS.

Multiple linear regression tested the unique contribution of each M-SES dimension to suicidal ideation severity.

Mediation analysis (general linear model) tested whether emptiness domain mediated the relationship between PID-5 domains and suicidal ideation (PID-5 domains → M-SES interpersonal → C-SSRS), with bootstrapped confidence intervals for indirect effects.

## 4 Results

### 4.1 Descriptive analysis

A total of 207 participants were initially recruited. Five participants did not provide consent for data use, resulting in a final sample of 202 individuals (71.3% female, 28.7% male), aged between 20 and 78 years (*M* = 41.2, *SD* = 14.4).

Most participants had a university-level education, with 49.5% holding a master’s degree, 29.2% a Ph.D. or equivalent advanced training, and 9.9% a bachelor’s degree; 11.4% completed secondary education. Regarding marital status, 60.9% were single, 27.2% married or cohabiting, 10.4% divorced, and 1.5% widowed. The majority (75.5%) reported being currently in a romantic relationship. With respect to health-related variables, 28.2% reported current use of medication.

34.7% reported current use of alcohol or other substances. Additionally, 32.2% indicated family history of psychiatric disorders.

Regarding indicators of suicidality assessed by the Columbia Suicide Severity Rating Scale (C-SSRS), 10.9% of participants reported suicidal ideation, 1% reported suicidal intent, and 5.3% reported a previous suicide attempt. Only two participants (1%) reported having thought about specific methods for suicide. As expected for a non-clinical population, the prevalence of suicidal behavior was low; therefore, subsequent analyses focused on the severity of suicidal ideation as the main indicator of suicidality.

Descriptive statistics for PID-5-SF domains and BSI subscales are reported in Table 1. Skewness and kurtosis values for all study variables fell within acceptable limits for normality (| skewness| < 2; | kurtosis| < 7), according to established guidelines, supporting the use of parametric analyses (Kim, 2013; Kline, 2023). As expected in non-clinical samples, all domains showed positive skewness, suggesting a low overall presence of pathological traits.

**TABLE 1:** PID-5-SF and BSI descriptive statistics.

Variables	Mean	Standard deviation	Skewness	Kurtosis
PID-5-SF				
Negative affectivity	0.660	0.592	0.981	0.255
Detachment	0.391	0.447	1.45	2.06
Antagonism	0.284	0.368	1.96	4.74
Disinhibition	0.462	0.466	1.07	0.706
Psychoticism	0.321	0.373	1.75	3.96
BSI				
GSI	29.7	27.3	1.60	2.79
Somatization	2.67	3.29	1.80	3.53
Obsession-compulsion	4.92	4.37	1.33	1.90
Interpersonal sensitivity	2.49	2.94	1.52	1.89
Depression	4.11	4.23	1.55	2.34
Anxiety	4.96	4.36	1.40	1.75
Hostility	2.62	3.21	2.35	6.83
Phobic anxiety	0.990	1.86	2.72	7.93
Paranoid ideation	2.66	3.13	1.64	2.58
Psychoticism	2.14	2.70	1.59	2.13

GSI, global severity index.

### 4.2 Statistic analysis

#### 4.2.1 Objective 1: M-SES structure and validity

An exploratory principal component analysis (PCA) was first conducted to examine the latent structure of the Modified Subjective Emptiness Scale (M-SES). The analysis suggested a four-factor solution, explaining 59.5% of the total variance. However, some items loaded on multiple components. Given the strong theoretical and clinical foundations underlying the multidimensional conceptualization of subjective emptiness, a confirmatory factor analysis (CFA) was subsequently performed using maximum likelihood estimation to test the hypothesized four-factor model.

Based on preliminary analysis of item performance, items 2 (λ = 0.438) and 7 (λ = 0.318) were removed due to weak loadings. The resulting 25-item model comprised the following latent factors:

(1) Physical/Bodily emptiness (items 1, 5, 8, 11, 14, 20, 25),
(2) Interpersonal/Social disconnection (items 3, 9, 12, 15, 18, 21, 24, 27),
(3) Dissatisfaction/Lack of purpose (items 6, 17, 23), and
(4) Severity/Self-level impairment (items 4, 10, 13, 16, 19, 22, 26).

The four-factor CFA model demonstrated a satisfactory fit to the data, χ^2^ (246) = 527, *p* < 0.001, CFI = 0.897, TLI = 0.884, RMSEA = 0.075 [0.066–0.084]. All items loaded significantly on their respective factors (all *p*s < 0.001), with standardized loadings ranging from 0.55 to 0.86, supporting the internal coherence of each subscale.

Inter-factor correlations were strong (*r* = 0.88–0.95, all *p*s < 0.001), suggesting that the four dimensions were closely related manifestations of a higher-order construct. To verify this hierarchical structure, a second-order CFA was conducted, testing whether the four M-SES domains loaded onto a general Subjective Emptiness factor. The second-order model showed an excellent fit, χ^2^ (2) = 0.18, *p* = 0.913, CFI = 1.00, TLI = 1.01, RMSEA = 0.00 [0.00–0.05]. All four domains loaded significantly on the higher-order factor (*p*s < 0.001), with standardized loadings ranging from 0.85 to 0.89. This hierarchical solution confirmed that subjective emptiness can be reliably conceptualized as a multidimensional yet unified construct encompassing bodily, interpersonal, existential, and structural disturbances of self-experience. Tables 2, 3 summarize these results.

**TABLE 2:** Confirmatory factor analysis (CFA) of the Modified Subjective Emptiness Scale (M-SES).

Factor	Item no.	Estimate	SE	*Z*	*P*	Std. estimate
Physical/bodily emptiness	1	0.707	0.0629	11.23	< 0.001	0.697
	5	0.803	0.0631	12.73	<0.001	0.764
	8	0.725	0.0635	11.43	<0.001	0.713
	11	0.561	0.0545	10.30	<0.001	0.676
	14	0.680	0.0672	10.12	<0.001	0.677
	20	0.783	0.0523	14.99	<0.001	0.865
	25	0.506	0.0450	11.25	<0.001	0.707
Interpersonal/social disconnection	3	0.563	0.0633	8.90	<0.001	0.591
	9	0.812	0.0752	10.80	< 0.001	0.687
	12	0.781	0.0610	12.81	< 0.001	0.775
	15	0.666	0.0617	10.80	< 0.001	0.687
	18	0.736	0.0569	12.93	< 0.001	0.781
	21	0.718	0.0629	11.41	< 0.001	0.717
	24	0.736	0.0765	9.62	< 0.001	0.628
	27	0.631	0.0748	8.43	< 0.001	0.564
Dissatisfaction/lack of purpose	6	0.790	0.0654	12.08	< 0.001	0.752
	17	0.971	0.0732	13.27	< 0.001	0.807
	23	1.031	0.0786	13.12	< 0.001	0.798
Severity/self-level impairment	4	0.666	0.0630	10.58	< 0.001	0.675
	10	0.709	0.0605	11.73	< 0.001	0.741
	13	0.549	0.0630	8.71	< 0.001	0.582
	16	0.679	0.0759	8.94	< 0.001	0.596
	19	0.588	0.0537	10.95	<0.001	0.696
	22	0.621	0.0779	7.96	<0.001	0.546
	26	0.656	0.0591	11.10	<0.001	0.707

**TABLE 3:** Second-order confirmatory factor analysis of the M-SES.

Indicator (M-SES subscale)	Estimate	SE	*Z*	*p*	Std. estimate
Interpersonal/Social disconnection	4.85	0.30	16.2	<0.001	0.89
Dissatisfaction/Lack of purpose	2.60	0.17	14.9	<0.001	0.85
Severity/Self-level impairment	4.34	0.27	16.0	<0.001	0.89
Physical/Bodily emptiness	4.46	0.28	16.0	<0.001	0.89

Higher-order factor: subjective emptiness.

Internal consistency of the Modified Subjective Emptiness Scale (M-SES) was examined using Cronbach’s α and McDonald’s ω. The overall scale showed excellent reliability (α = 0.917, ω = 0.930). All subdimensions demonstrated high internal consistency, with α coefficients ranging from 0.879 to 0.916 and ω coefficients from 0.905 to 0.917. Table 4 summarizes the reliability indices for the total scale and its four subdimensions.

**TABLE 4:** Internal consistency of the M-SES and subscales.

Subscale	Cronbach’s α	McDonald’s ω
M-SES physical/bodily emptiness	0.882	0.907
M-SES interpersonal/social disconnection	0.884	0.905
M-SES dissatisfaction/lack of purpose	0.916	0.917
M-SES severity/self-level impairment	0.879	0.906
M-SES total scale	0.917	0.930

The average inter-item correlations (AICs) estimated from Cronbach’s α ranged between 0.44 and 0.48 across all subscales, indicating good internal coherence. The Dissatisfaction/Lack of Purpose factor showed a slightly higher AIC (0.62), indicating a possible overlap.

The four M-SES subscales were strongly intercorrelated (*r* = 0.75–0.79, *p* < 0.001), suggesting that they capture related yet distinct domains of the subjective experience of emptiness.

Regarding convergent validity with general psychological distress, strong positive correlations emerged between all M-SES dimensions and the Global Severity Index (GSI) of the Brief Symptom Inventory (*r* = 0.66–0.72, *p* < 0.001), supporting the convergent validity of the scale. Higher levels of subjective emptiness were associated with increased depressive symptoms, anxiety, and interpersonal sensitivity, indicating that emptiness reflects a pervasive affective and relational disturbance. Moderate associations with somatization and hostility suggest that emptiness primarily captures internalized psychological suffering rather than externalizing or somatic distress. Strong correlations with paranoid ideation and psychoticism further support the link between emptiness and disturbances in self-experience and identity continuity (see Table 5).

**TABLE 5:** Pearson’s correlations between BSI symptom dimensions and M-SES subscales.

BSI dimension	M-SES physical/bodily	M-SES interpersonal/social disconnection	M-SES dissatisfaction/lack of purpose	M-SES severity/ self-level disturbance
Global severity index	0.68***	0.69***	0.67***	0.75***
Somatization	0.42***	0.42***	0.40***	0.50***
Obsessive–compulsive	0.66***	0.61***	0.63***	0.66***
Interpersonal sensitivity	0.60***	0.70***	0.63***	0.71***
Depression	0.72***	0.68***	0.66***	0.67***
Anxiety	0.55***	0.55***	0.57***	0.64***
Hostility	0.54***	0.49***	0.48***	0.58***
Phobic anxiety	0.51***	0.60***	0.49***	0.58***
Paranoid ideation	0.52***	0.60***	0.54***	0.62***
Psychoticism	0.64***	0.70***	0.63***	0.67***

***p < 0.001. BSI, Brief Symptom Inventory; M-SES, Modified Subjective Emptiness Scale.

#### 4.2.2 Objective 2: exploring the associations between PID-5; CSS-RS; M-SES

Pearson’s correlations were computed to examine the associations between the M-SES subscales, maladaptive personality traits (PID-5-SF), and suicidal ideation severity (C-SSRS). This allow both to expand considerations on external validity of the M-SES and to begin to explore the relationship between focus variables. Pearson’s correlations between the M-SES subscales and PID-5-SF domains supported the convergent and discriminant validity of the scale (see Table 6). All M-SES dimensions showed strong positive associations with Negative Affectivity (*r* = 0.46–0.59, *p* < 0.001) and Detachment (*r* = 0.45–0.56, *p* < 0.001), indicating that feelings of emptiness are closely linked to affective dysregulation and interpersonal withdrawal. Moderate correlations with Psychoticism (*r* = 0.23–0.44, *p* < 0.001) further support the connection between emptiness and disturbances in self-experience. In contrast, nonsignificant or weak correlations were observed with Antagonism (*r* = 0.10–0.27, *p* < 0.05), consistent with the conceptualization of emptiness as primarily an internalizing rather than externalizing phenomenon. Among the subscales, the Severity/Self-level disturbance dimension exhibited the strongest associations across all domains, highlighting its central role as an indicator of global personality dysfunction. All M-SES dimensions except for Dissatisfaction/Lack of Purpose showed positive correlations with suicidal ideation severity (*r* = 0.21–0.23, *p* ≤ 0.01), supporting the criterial validity of the M-SES in capturing affective pain implicated in suicidal processes.

**TABLE 6:** Pearson’s correlations between M-SES subscales, PID-5-SF personality domains, and suicidal ideation severity (C-SSRS).

PID-5-SF	M-SES physical/bodily	M-SES interpersonal/social disconnection	M-SES dissatisfaction/lack of purpose	M-SES severity/self-level disturbance
Negative affectivity	0.49***	0.51***	0.46***	0.59***
Detachment	0.54***	0.56***	0.45***	0.50***
Antagonism	0.19**	0.17*	0.10	0.27***
Disinhibition	0.46***	0.45***	0.36***	0.57***
Psychoticism	0.32***	0.36***	0.23***	0.44***
Suicidal ideation severity	0.21**	0.23***	0.11	0.21**

*p < 0.05,

**p < 0.01,

***p < 0.001.

#### 4.2.3 Objective 3: mediation model

Previous to the mediation model, a multiple linear regression analysis was conducted to examine whether pathological personality traits and dimensions of subjective emptiness predicted suicidal ideation severity (C-SSRS), controlling for age and gender.

The overall model was significant, *F*(8, 192) = 2.53, *p* = 0.012, explaining 9.5% of the variance in suicidal ideation severity (*R*^2^ = 0.095). Among predictors, the M-SES Interpersonal/Social Disconnection dimension was the only significant predictor (β = 0.21, *p* = 0.041), indicating that higher levels of interpersonal emptiness were associated with greater suicidal ideation severity. Collinearity diagnostics indicated acceptable tolerance levels (all VIFs < 3.5).

At this point a series of mediation models were conducted to examine whether the Interpersonal/Social Disconnection dimension of the M-SES mediated the relationships between pathological personality traits (PID-5-SF) and suicidal ideation severity (C-SSRS).

Only indirect effects were significant for Detachment (β = 0.097, *p* = 0.040) and Psychoticism (β = 0.057, *p* = 0.044). The mediation for Negative Affectivity did not reach conventional significance (β = 0.078, *p* = 0.058), although the total effect was significant (β = 0.211, *p* = 0.002). For Disinhibition, only the direct effect remained significant (β = 0.193, *p* = 0.011), while indirect effect did not reached significance (β = 0.060, *p* = 0.083). These results are summarized in Table 7.

**TABLE 7:** Indirect, direct, and total effects of PID-5 domains on suicidal ideation severity, mediated by interpersonal/social disconnection (M-SES).

Predictor (PID-5 domain)	Effects	β	SE	*z*	*p*
Negative affectivity	Indirect	0.078	0.029	1.89	0.058
	Direct	0.133	0.056	1.68	0.092
	Total	0.211	0.049	3.05	0.002
Detachment	Indirect	0.097	0.044	2.05	0.040
	Direct	0.085	0.078	1.03	0.301
	Total	0.182	0.065	2.63	0.009
Disinhibition	Indirect	0.060	0.031	1.74	0.083
	Direct	0.193	0.068	2.56	0.011
	Total	0.253	0.061	3.71	<0.001
Psychoticism	Indirect	0.057	0.032	2.02	0.044
	Direct	0.177	0.082	2.44	0.015
	Total	0.233	0.077	3.40	<0.001

## 5 Discussion

The present study aimed to develop and validate the Modified Subjective Emptiness Scale (M-SES) in order to investigate the associations between subjective emptiness, pathological personality traits, and suicidal ideation severity in a general population sample. The findings support the multidimensional structure, internal consistency, and theoretical validity of the M-SES. Moreover, the results provide empirical evidence for the role of interpersonal emptiness as painful affective experience linking maladaptive personality traits to suicidal ideation.

### 5.1 M-SES structure and validity

The confirmatory factor analysis confirmed the fit of the hypothesized four-factor structure 1. Physical/Bodily emptiness, 2. Interpersonal/Social disconnection, 3. Dissatisfaction/Lack of purpose, and 4. Severity/Self-level impairment. This structure mirrors the multidimensional conceptualization of emptiness proposed in recent clinical and theoretical work (D’Agostino et al., 2020; Hudson et al., 2024). The confirmatory analysis was chosen to address for both the strong theoretical framework and the non-clinical sample that did not allow for data driven-only analysis. Moreover, a higher-order general emptiness factor was discovered, suggesting that the four dimensions represent different facets of a general disturbance in emotional regulation and self-experience. In fact, this vision aligns with the disruption in the pre-reflective sense of self-presence and vitality (Parnas et al., 2005; Stephensen et al., 2024) and with the notion of emptiness as an affective marker of identity diffusion and weakened representational continuity (Kernberg, 1975). Subjective emptiness emerged as both a multidimensional and hierarchically integrated construct: such a result addresses previous limitations of the SES-I, which measured emptiness only as unidimensional.

### 5.2 Exploring the associations between PID-5; CSS-RS; M-SES

All dimensions of the M-SES were positively strongly associated with Negative Affectivity and Detachment, in line with previous studies (Konjusha et al., 2021) and moderately correlated with Psychoticism. These findings indicate that subjective emptiness reflects affective dysregulation, interpersonal withdrawal, and fragmentation of self-experience, core features of maladaptive personality functioning that can be captured in the dimensional AMPD model (Hopwood and Gjorgjieva, 2024; Hudson et al., 2024; Sharp and Wall, 2021). The correlations reveal clinically meaningful patterns. The Physical/Bodily emptiness dimension showed the strongest association with Detachment, suggesting that the loss of inner vitality and sensory connectedness may represent the experiential counterpart of emotional numbing and social disengagement (Ellison et al., 2016; Zandersen and Parnas, 2019). Previous studies point to the close correspondence between detachment and low positive affectivity rather than high negative affectivity (Watson et al., 2019), which is consistent with the interpretation of bodily emptiness as an absence of embodied feeling where individuals experience a disconnection from their own visceral affective life (Klonsky, 2008; Konjusha et al., 2021). For the Interpersonal/Social disconnection dimension the strongest correlation was also Detachment, in line with its focus on relational alienation and perceived distance from others (Heinrich and Gullone, 2006). In contrast, the Dissatisfaction/Lack of Purpose factor showed the strongest association with Negative Affectivity, reflecting its affective tone of despair, dysphoria, and hopelessness. This pattern appears to capture the depressive dimension of emptiness, characterized by a pervasive loss of meaning and purpose and hopelessness (Hudson et al., 2024; Tanzilli et al., 2021). Consistent with this interpretation, Hudson et al. (2024) identified purposelessness as the most frequently reported emptiness dimension among individuals with depressive disorders, with more than 30% endorsing this feature. Severity/Self-level impairment factor also showed the strongest association with Negative Affectivity, supporting the notion that when the experience of emptiness becomes rooted in a disturbance of the basic self, it is accompanied by intense affective suffering (Parnas et al., 2005; Stephensen et al., 2024). This finding suggests that the more the disturbance involves the basic level of self, the more emptiness is experienced as an anguishing, overwhelming state. In this sense, the affective tone of Negative Affectivity may represent the emotional correlate of a deeper disorganization of subjectivity. Notably, the Severity/Self-level impairment domain displayed the broadest and strongest associations across all PID-5 domains, underscoring its role as a central marker of global personality dysfunction and structural disturbance of the self.

Finally, consistent with prior evidence linking emptiness to suicidality (Konjusha et al., 2021; Meddaoui et al., 2025), all M-SES dimensions except Dissatisfaction/Lack of Purpose correlated positively with suicidal ideation severity. This supports the criterial validity of the scale and suggests that feelings of relational disconnection and internal void may serve as emotional trigger through which personality vulnerability translates into suicidal thoughts.

### 5.3 Mediation models

Building on correlation and regression findings, the mediation analyses were designed to clarify the role of the interpersonal dimension of subjective emptiness as potentially linking maladaptive personality traits to suicidal ideation. This approach was guided by theoretical and empirical models positing that affective and relational disturbances mediate the progression from personality vulnerability to suicidal thinking (Casini et al., 2024; Meddaoui et al., 2025).

Among the M-SES domains, Interpersonal/Social Disconnection emerged as the only significant predictor of suicidal ideation severity in the regression model, in line with clinical and empirical literature suggesting that feelings of isolation, loneliness, deprived relationships, are particularly relevant in the suicidal process (Casini et al., 2024; Gürcan et al., 2023; Motillon-Toudic et al., 2022; Williams et al., 2025). These findings may be more fully understood within the framework of the Interpersonal Psychological Theory of Suicide (IPTS, Van Orden et al., 2010), which posits that suicidal ideation primarily arises from unmet interpersonal needs, particularly thwarted belongingness and perceived burdensomeness. From this perspective, the interpersonal dimension of emptiness may represent the affective experience of relational disconnection underlying suicidal suffering. Rather than reflecting loneliness alone, interpersonal emptiness may involve a deeper sense that emotional bonds with others are inaccessible, devoid of meaning, or irreparably lost.

In the mediation models, significant indirect effects were found for Detachment and Psychoticism via Interpersonal Disconnection, while Negative Affectivity approached significance. These findings, in line with IPTS, indicate that personality traits characterized by emotional withdrawal and disturbances in self–other boundaries contribute to suicidal ideation primarily through their impact on the experience of interpersonal emptiness. Such a lived experience may create fertile ground for suicidal ideation given that interpersonal connection is consistently recognized as one of the most powerful protective factors against suicide (Ho Choi et al., 2013). Clinically, this pattern highlights that it is not simply the presence of negative affect or social withdrawal that could underlie suicidal ideation, but the subjective internalization of experiencing relations as impossible or lacking in meaning, leading to a state of profound disconnection from others and from oneself. Interpersonal emptiness may thus function as an emotional bridge between structural personality vulnerabilities and the emergence of suicidal thoughts, a process consistent with phenomenological accounts describing emptiness as the experiential core of alienation and self-fragmentation (Parnas et al., 2005; Stephensen et al., 2024; Zandersen and Parnas, 2019).

For Psychoticism, both direct and indirect paths were significant. The direct path is in line with current literature (Yates et al., 2019). The indirect pathway linking Psychoticism to suicidal ideation via Interpersonal Disconnection is in line with this interpretation of altered self–world demarcation and loss of first-personal coherence in exacerbating feelings of alienation. In such cases, the feeling of unreality and fragmentation may render the experience of emptiness particularly unbearable, intensifying suicidal ideation as an attempt to restore coherence and self-presence. This result can help to interpret studies that link some aspects of psychotic experience (e.g., persecutory ideation) but not others (e.g., bizarre experiences) with suicidal ideation (Capra et al., 2015).

For Detachment, only the indirect effect was significant: the severity of suicidal ideation does not seem to depend on detachment per se, but rather on its transformation into an inability to form meaningful bonds. In other words, detachment becomes clinically dangerous when it crystallizes into alienation, leading to an interpersonal void that fuels more intense suicidal ideation. Individuals high in detachment may experience a progressive erosion of interpersonal meaning and belonging, and suicidal thoughts may emerge as an attempt to counteract or escape this unbearable sense of disconnection. This result is in line with studies indicating interpersonal motivation as primarily connected to suicidal risk (Casini et al., 2024).

For Negative Affectivity, the indirect pathway through interpersonal disconnection approached significance (*p* = 0.058). This finding suggests that chronic emotional suffering and dysphoria may constitute a general background of vulnerability to suicidality (D’Agostino et al., 2018; Overholser et al., 2023; Shneidman, 1993), rather than a process-specific driver. Negative Affectivity might represent a diffuse affective terrain of distress, broadly associated with suicidal vulnerability but not uniquely characterizing the suicidal process itself. In some individuals, however, this suffering takes the form of profound loneliness and inner emptiness, which may explain the emerging mediating trend of the emptiness construct in this pathway.

Finally, coherently with clinical considerations, for Disinhibition only the direct effect on suicidal ideation severity was significant. Disinhibition is confirmed to enhances the likelihood of more intense action-like suicidal thoughts, through dysregulation and impulsive cognition (Allen et al., 2022).

## 6 Conclusion

The present study represents an attempt to validate a multidimensional instrument specifically designed to assess subjective emptiness in its phenomenological complexity and to investigate its role in personality functioning and suicidal ideation. The results support a four-factor hierarchical model of emptiness, confirming its internal coherence and strong convergent validity with both maladaptive personality traits and general psychopathological distress.

A major strength of this study lies in its integration of phenomenological and psychodynamic perspectives within an empirically grounded framework, offering a bridge between clinical psychopathology, dimensional models of personality and empirical work. However, several limitations should be acknowledged. First, the cross-sectional design prevents causal inferences regarding the temporal relationships among the variables. Second, the study was conducted on a non-clinical sample, whereas the M-SES was conceptually developed to assess clinical dimensions of subjective emptiness. Although this approach allowed for an initial exploratory validation of the scale, further research is needed to confirm its structure, validity, and clinical sensitivity in clinical samples. The present findings should therefore be interpreted as preliminary, providing the empirical groundwork for future studies in clinical populations.

Moreover, the strong associations between the M-SES dimensions and BSI indices, particularly depressive symptoms and global psychological distress, suggest that subjective emptiness and depression are closely related but not equivalent constructs. Rather than indicating redundancy, this overlap may reflect the presence of partially shared affective and phenomenological features, while emptiness retains clinically meaningful specificity in its bodily, interpersonal, existential, and self-structural manifestations. In particular, the finding that Interpersonal/Social Disconnection, but not Dissatisfaction/Lack of Purpose, uniquely predicted suicidal ideation suggests that the suicidal relevance of emptiness cannot be reduced to depressive hopelessness or dysphoria alone.

Despite these limitations, data suggest that emptiness does play a significant role in the suicidal process, particularly in its interpersonal dimension. This is not a mere “feeling of loneliness,” but rather an experience of relational rupture, a perception that bonds with others have lost meaning, have become inaccessible, or are irreparably broken. It represents a form of emptiness that undermines the very foundations of belonging and recognition, fostering the conviction of having no place among others. It is the capacity to feel that relationships are possible, that connection can be maintained and reactivated, that grants the individual the certainty of not being irredeemably alone. When this internal representation is fragile or absent, what remains is an absolute void. In this condition, suicide may emerge as an extreme attempt to manage or escape from this inner emptiness.

## Data Availability

The raw data supporting the conclusions of this article will be made available by the authors, without undue reservation.

## Footnotes

1
https://cssrs.columbia.edu/the-columbia-scale-c-ssrs/translations/
